# Acceleration of bone formation by octacalcium phosphate composite in a rat tibia critical-sized defect

**DOI:** 10.1016/j.jot.2022.09.007

**Published:** 2022-10-12

**Authors:** Cheol-Hee Jeong, Jooseong Kim, Hyun Sil Kim, Song-Yi Lim, Dawool Han, Aaron J. Huser, Sang Bae Lee, Yeonji Gim, Jeong Hyun Ji, Dohun Kim, Amaal M. Aldosari, Kyelim Yun, Yoon Hae Kwak

**Affiliations:** a Department of Oral Pathology, Yonsei University College of Dentistry, Seoul, South Korea; b Department of Biomedical Engineering, Yeungnam University, Daegu, Republic of Korea; c HudensBio Co., Ltd., Gwangju, Republic of Korea; d Department of Orthopedic Surgery, Asan Medical Center, Ulsan University College of Medicine, Seoul, South Korea; e Paley Advanced Limb Lengthening Institutute, St. Mary’s Hospital, West Palm Beach, FL, USA; f Center for Testing and Evaluation of Dental Biomaterials, Ministry of Food and Drug Safety Recognition Laboratory, Yonsei University College of Dentistry, Seoul, South Korea; g Department of Laboratory Animal Resources, Yonsei Biomedical Research Institute, Yonsei University College of Medicine, Seoul, South Korea; h Department of Orthopedic Surgery, Al Noor Specialist Hospital, Makkah, Saudi Arabia

**Keywords:** Octacalcium phosphate, β-tricalcium phosphate, Bone regeneration, Bone substitute, Critical-sized defect

## Abstract

**Background:**

The osteogenic capabilities and biodegradability of octacalcium phosphate (OCP) composites make them unique. Despite the excellent characteristics of OCP, their use is limited due to handling difficulties. In this study, we aimed to evaluate and compare three types of OCPs (cemented OCP (C-OCP), C-OCP with collagen (OCP/Col), and synthetic OCP (S-OCP) with alginate (OCP/Alg)) versus commercially available β-tricalcium phosphate (β-TCP) regarding their potential to accelerate bone formation in defective rat tibias.

**Methods:**

The specimens with OCP composite were manufactured into 5 ​mm cubes and inserted into the segmental defects of rat tibias fixed with an external fixator. In addition, 3 ​mm-hole defects in rat tibias were evaluated to compare the graft material properties in different clinical situations. Serial X-ray studies were evaluated weekly and the tibias were harvested at postoperative 6 weeks or 8 weeks for radiologic evaluation. Histological and histomorphometric analyses were performed to evaluate the acceleration of bone formation.

**Results:**

In the critical-defect model, OCP/Alg showed bone bridges between segmentally resected bone ends that were comparable to those of β-TCP. However, differences were observed in the residual graft materials. Most β-TCP was maintained until 8 weeks postoperatively; however, OCP/Alg was more biodegradable. In addition calcification in the β-TCP occurred at the directly contacted area between graft particles and bony ingrowth was observed in the region adjacent resected surface of tibia. In contrast, no direct bony ingrowth was observed in OCP-based materials, but osteogenesis induced from resected surface of tibia was more active. In the hole-defect model, OCP/Col accelerated bone formation. β-TCP and OCP/Alg showed similar patterns with relatively higher biodegradability. In histology, among the OCP-based materials, directly contacted new bone was formed only in OCP/Alg group. The new bone formation in the periphery area of graft materials was much more active in the OCP-based materials, and the newly formed bone showed a thicker trabecular and more mature appearance than the β-TCP group.

**Conclusions:**

In this study, OCP/Alg was equivalent to β-TCP in the acceleration of bone formation with better biodegradability appropriate for clinical situations in different circumstances. Our OCP/Col composite showed fast degradation, which makes it unsuitable for use in mechanical stress conditions in clinical orthopedic settings.

**The Translational Potential of this Article:**

In our research, we compared our various manufactured OCP composites to commercially available β-TCP in critical-defect rat tibia model. OCP/Col showed acceleration in hole-defect model as previous studies in dental field but in our critical-sized defect model it resorbed fast without acceleration of bony union. OCP/Alg showed matched results compared to β-TCP and relatively fast resorption so we showed market value in special clinical indication depending on treatment strategy. This is the first OCP composite study in orthopaedics with animal critical-sized tibia bone study and further study should be considered for clinical application based on this study.

## 1 Introduction

Spontaneous bone healing after fracture is a proliferative physiological process in which the body facilitates repair. The process of fracture healing requires a precise balance between biology and stabilization; the four pillars of adequate bone healing are mechanics, osteogenic cells, scaffolds, and growth factors [1]. In situations where bone defects are present, where the fracture does not heal spontaneously, bone grafting is an option to salvage the extremity [2]. The selection of an appropriate bone graft material is essential for satisfactory and sufficient bone growth to achieve successful bony union [3,4]. An ideal bone graft substitute should be cost-effective and ultimately encourage new bone formation based on its physiology, biocompatibility, and biodegradability [5].

The physiology of bone grafts is well-defined and includes osteoconductivity, osteoinductivity, and osteogenesis [6,7]. Autologous bone is an ideal physiologic graft material because it can have all three properties; however, it is limited by the volume that can be obtained and donor-site morbidity [3]. Allografts are osteoconductive because they offer scaffolds for new bone to build upon, and some, such as demineralized bone matrices, may also have osteoinductive properties [6]. Additionally, biological growth factors, such as bone morphogenic proteins, have been developed and studied for their role as osteoinductive agents [6]. Synthetic osteoconductive scaffolds also play an important role in the reconstruction of bone defects; examples include hydroxyapatite (HA) and β-TCP. One of the most important properties of these scaffolds is the rate at which they degrade compared to the rate at which new bone forms [8,9]. Calcium phosphate materials are known to degrade at a rate closer to that of bone formation than HA at physiological pH [10]. β-TCP is safely and effectively used in clinical settings as an osteoconductive scaffold; however, it does not naturally contain growth factors or osteogenic cells [11].

Based on previous studies, OCP has emerged as an alternative synthetic scaffold with osteoinductive properties and an improved biodegradability profile over β-TCP [12,13]. In animal models, OCP has been shown to accelerate intramembranous and enchondral ossification [14]. Higher expression of ALP, RUNX2, and OSX genes by OCP suggests greater potential of other biomaterials for induction of differentiation of SCAPs to osteoblasts [15]. In addition, OCP is a precursor of HA [16]. Previous studies have investigated OCP use in the calvaria, mandible, and frontal bones, which has led to the approval of commercially available products for use in dentistry [[17], [18], [19], [20]]. OCP studies in the lower extremities have previously examined tibial bone hole defects and critical-sized defects in a rat model; however, none have investigated alginate or collagen composites [10,[21], [22], [23]]. Notably, OCP composites have not been approved for orthopedic use because of the paucity of preclinical studies.

Collagen is a cell-attaching matrix protein, and its spongy nature has been widely used as a scaffold [[18], [19], [20],[24], [25], [26]]. OCP/Col generates osteoids, osteoclasts, and microvessels, signifying true bone formation mimicking bone tissue; however, the exact origin of the regenerated bone is yet to be determined. Alginate is a natural polysaccharide obtained from brown seaweed that forms relatively stable hydrogels through ionotropic gelation in the presence of divalent cations, with calcium ions being the most widely used [27]. Apart from its relatively high cost compared to that of other materials, alginate has several advantages, such as biocompatibility, biodegradability, and low immunogenicity. Thus, alginate has implantable properties in vivo and functions as a scaffold for various active substances, such as growth factors [28].

The purpose of this study was to compare β-TCP, C-OCP, and two OCP composites for the treatment of critical-sized tibial defects and tibial hole defects in a rat model. The OCP composites were cemented OCP with collagen (OCP/Col) and synthetic OCP with alginate (OCP/Alg). We also evaluated the biodegradability of the scaffolds relative to bone regeneration of the tibia. Our hypothesis was that OCP/Col and OCP/Alg would have faster healing and degradation than that of β-TCP and C-OCP.

## 2 Materials and methods

### 2.1 Preparation of OCP composite

**Figure 1:**
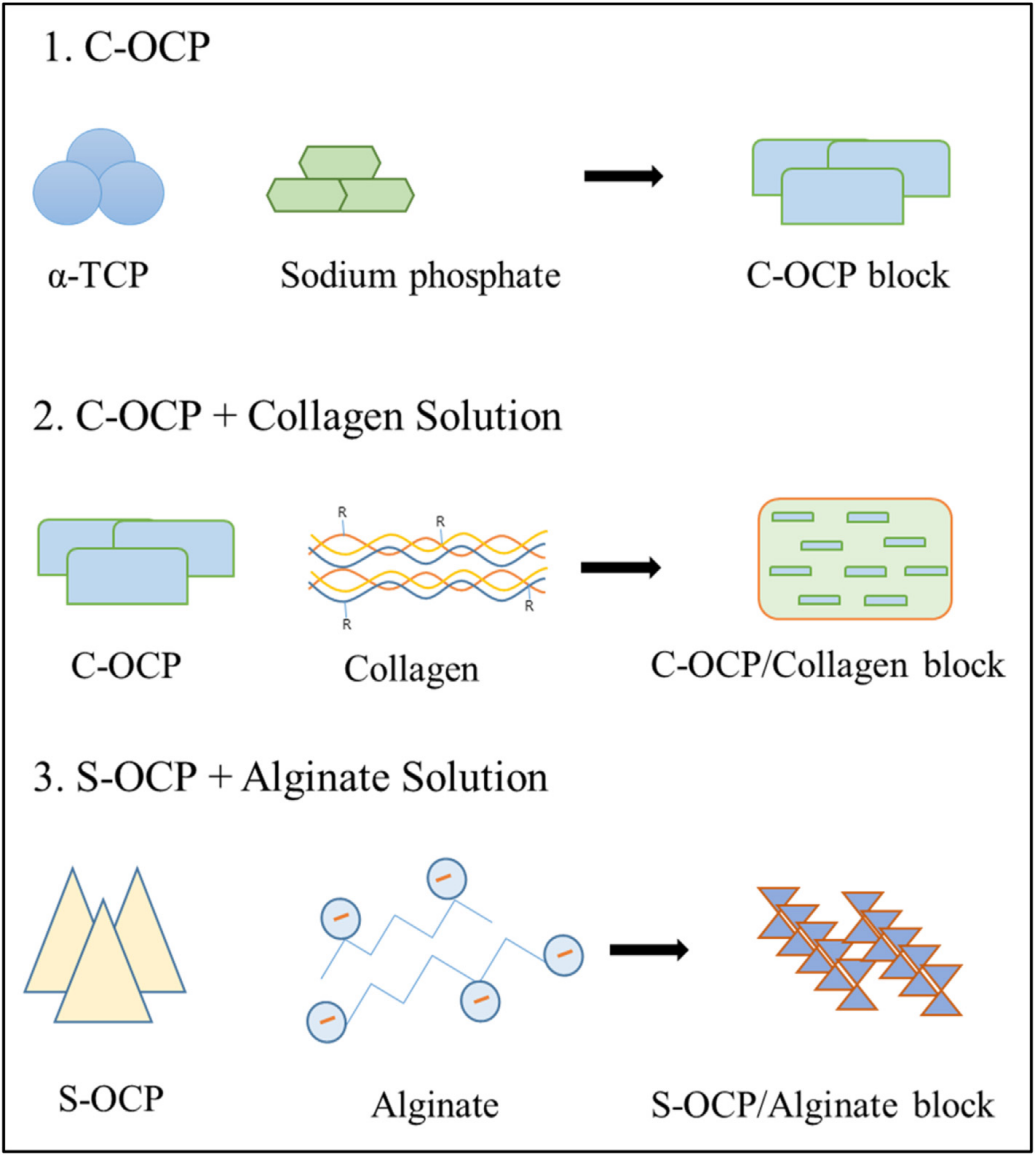
Schematic images showing the manufacturing process of (1)C-OCP, (2)OCP/Col, (3)OCP/Alg. β-TCP is a commercially available specimen and is therefore not described.

**Figure 2:**
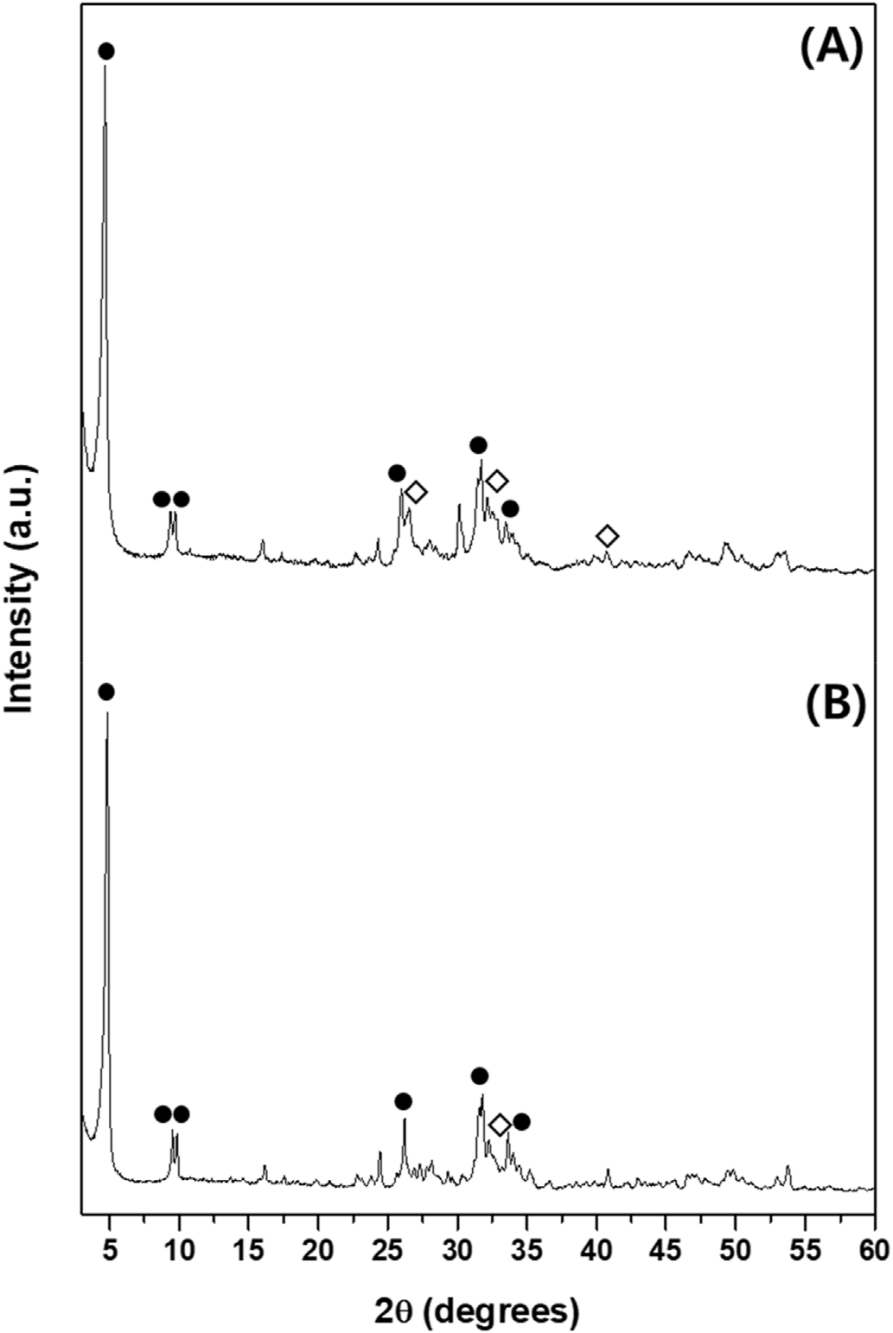
XRD patterns of the (A) C-OCP and (B) S-OCP; the OCP and HA peaks are indicated by • and ◇, respectively.

**Figure 3:**
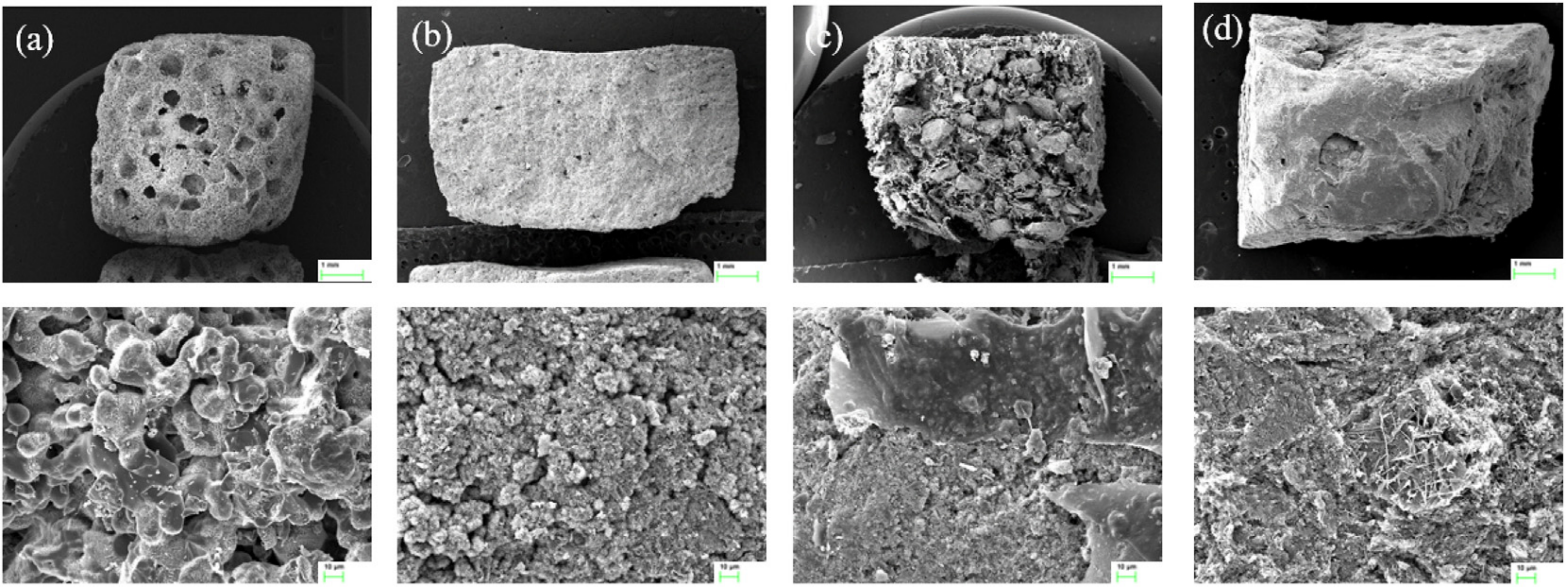
Observation by the scanning electron microscope. (a) β-TCP, (b) C-OCP, (c) OCP/Col, (d) OCP/Alg. Surface of material at the upper row was screened by 11x to 15x (scale bar ​= ​1 ​mm). Graft materials were manufactured to be 5-mm in height. Lower row shows 500x magnification (scale bar ​= ​10 ​μm).

A cemented OCP (C-OCP) sample was prepared by hydration of a mixture of α-TCP and sodium phosphate. The Ca/P ratio of the paste was 1.00 that was poured into a rectangular mold (20 ​× ​10 ​mm). The reaction was performed in a humidity chamber (Jeio Tech Co., Ltd, Korea) at 70 ​°C RH 95% for 2 ​h. The dried C-OCP on which the cement reaction had been completed was ground and sieved to a size of 500–850 ​μm. The C-OCP particles (500–850 ​μm) and 3 ​wt% collagen solution (collagen from porcine skin, Dalim Tissen Co., Ltd., Republic of Korea) were mixed and poured into a rectangular mold (20 ​× ​10 ​mm). The mixture was dried at −50 ​°C for one day using a freeze dryer (Ilshin Biobase Co., Ltd.). The dried samples were crosslinked in a vacuum oven (Jeio Tech Co., Ltd., Korea) at 120 ​°C for 16 ​h. To use smaller OCP particles (less than 50 ​μm), a synthetic OCP (S-OCP) sample was prepared by adjusting the pH of dicalcium phosphate dihydrate (DCPD) using acetic acid. The S-OCP powder was mixed with a 3 ​wt% alginate solution (Daejung Chemicals and Metals Co., Ltd., Republic of Korea) and poured into a rectangular mold (20 ​× ​10 ​mm). Subsequently, the mixture was dried at 25 ​°C for one day (Fig. 1). β-TCP (Neobone®; SNbiologics, Seoul, Korea) was commercially available. Fig. 2 shows the phase analysis of cemented and synthesized OCP and despite the addition of collagen and alginate, the OCP phase was well-formed and no impurities were detected within the XRD detection limit. The morphology was observed using a Zeiss Merlin analytical scanning electron microscope (SEM) (Fig. 3). The calculated density (g/cm^3^) of each sample was 3.14(β-TCP), 2.72(C-OCP), 2.68(OCP/Col) and 2.56(OCP/Alg).

### 2.2 Implantation of OCP composites into the critical-sized defect rat tibia

**Figure 4:**
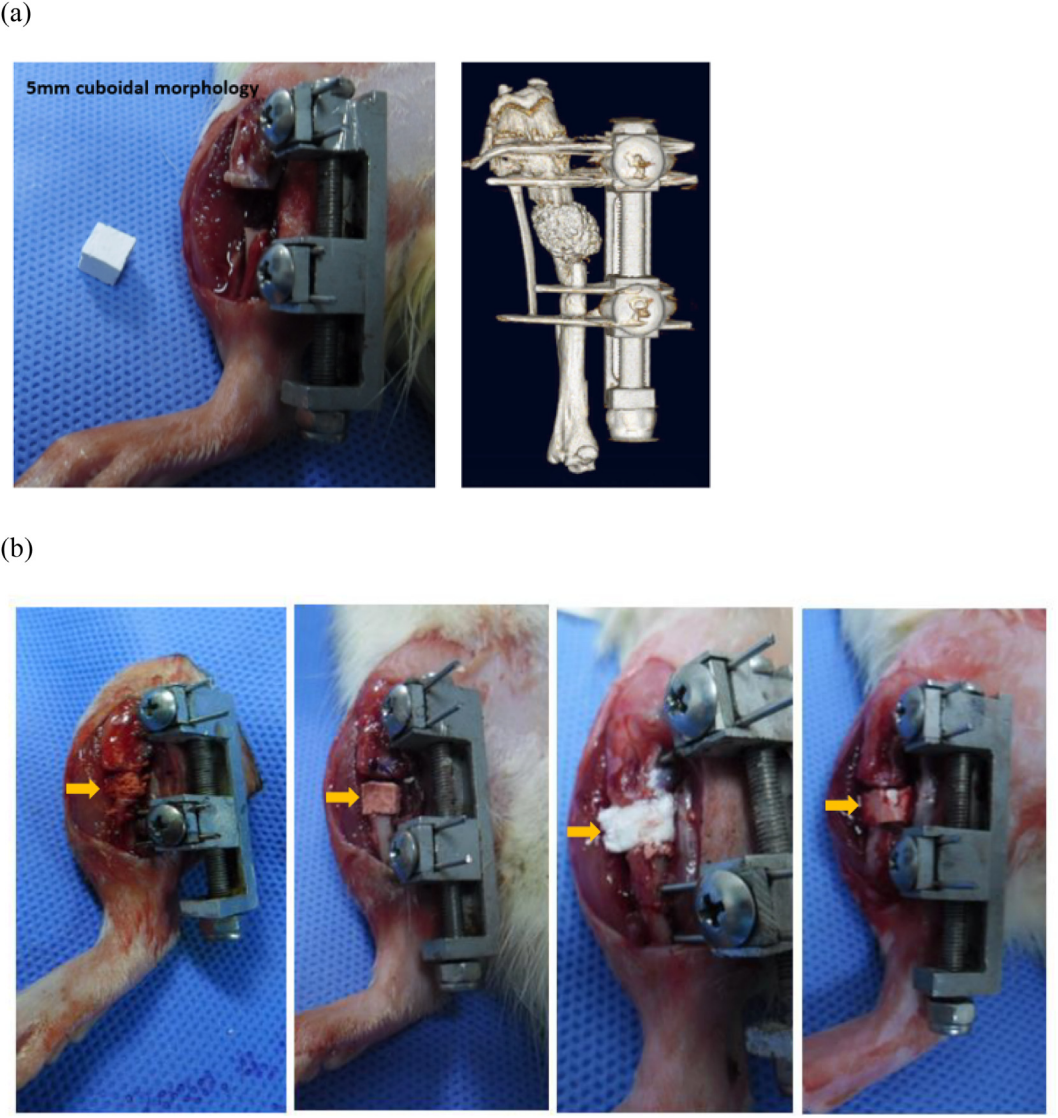
Surgery of critical bone defect of rat tibia. (a) After applying the external fixator (EF), a 5-mm defect of the rat tibia was made with a saw blade under continuous saline irrigation to prevent thermal damage. The fibula was broken manually. The defect was further distracted to insert a 4-6-mm sized graft material then firmly fixed by compressing the fixator. Manual testing was performed to ensure that the graft material was stable. 3D CT image showed surgical model after graft insertion. (b) Gross images after insertion of β-TCP, C-OCP, OCP/Col and OCP/Alg. β-TCP, C-OCP and OCP/Alg were firmly compressed between two bone ends. However, OCP/Col has relatively low stiffness and was unable to tolerate over-compression by the external fixator. Yellow arrows indicate implanted graft.

The experiments were performed in compliance with the principles of laboratory animal care, use, and national laws. Notably, 12-week-old Sprague Dawley rats (Orient Bio Co., Seoul, Korea) were used in this study and housed under a 12-h light, 12-h dark cycle. Food and water were provided ad libitum. A total of 24 male rats weighing 350–400 ​g were used for the critical-defect-sized rat tibial model [29]. 24 Rats were divided into four groups as beta-TCP, C-OCP, OCP/Col and OCP/Alg as 6 rats each. After the induction of anesthesia, the surgical area was shaved and disinfected. A 2 ​cm longitudinal incision was made anteriorly at the midshaft of the tibia. The tibialis anterior (TA) muscle was protected using a retractor. A 0.7 ​mm Kirschner's wire (K-wire, Zimmer Biomet, Warsaw, IN) was used as a drill bit and four 0.9 ​mm K-wires were inserted; two wires were placed proximal to the location of the proposed defect and two wires were placed distally. A custom-made external fixator/rail was attached to the wires and a 5 ​mm defect was created between the proximal and distal wires using a saw blade under saline irrigation to prevent thermal damage (Fig. 4(a)). After confirmation of a broken fibula, one of the 5-mm cuboidal synthetic scaffolds (β-TCP, C-OCP, OCP/Col, or OCP/Alg) was implanted (Fig. 4(b)). The external fixator was firmly tightened and the implant was confirmed to be in a stable position. The incision was subsequently closed layer-by-layer using sutures. Analgesia (meloxicam, 1 ​mg/kg) and antibiotics (enrofloxacin, 2.5 ​mg/kg) were administered to all rats until postoperative day three. All rats were permitted to move freely without immobilization immediately after surgery.

### 2.3 Implantation of OCP composites into the hole defect of rat tibia

A total of 30 male rats, weighing 350–400 ​g, were used for the tibial hole defect model. 30 Rats were divided into five groups as none, beta-TCP, C-OCP, OCP/Col and OCP/Alg as 6 rats each. After anesthesia induction, the surgical area was shaved and disinfected. A 1 ​cm longitudinal incision was made in the proximal one-third of the tibia. A 1.2-mm diameter K-wire was used to pre-drill the tibia, followed by a 3 ​mm drill under saline irrigation (Supplement figure S1). One of the synthetic scaffolds (β-TCP, C-OCP, OCP/Col, or OCP/Alg) was packed into the 3 ​mm-hole. The incision was closed in a layered manner. Analgesia (meloxicam 1 ​mg/kg) and antibiotics (enrofloxacin, 2.5 ​mg/kg) were administered to all rats during surgery. Bilateral tibias were used for the hole defect model [30]. All rats were permitted to move freely without immobilization immediately after surgery.

### 2.4 Radiograph and micro-CT analyses

The quality of bone formation was serially assessed using DEXA (Inalyzer, Medikors, Sungnam, Korea) under anesthesia. DEXA scans were performed immediately postoperatively, and at 1 week, 2 weeks, 4 weeks, 6 weeks, and 8 weeks. The rats were euthanized postoperatively at the 6 week or 8 week mark [29,31]. The tibias were fixed in 10% formalin for 48 ​h at room temperature and transferred to 70% ethanol. After fixation, the external fixator was removed from the tibia before scanning. Quantitative three-dimensional evaluation of new bone formation and bony union was performed using a micro-CT imaging system at 90 ​kVp and 88 ​μA with a resolution of 144 ​μm voxel size. The images (Quantum GX2, Perkin Elmer, Waltham, MA) were analyzed by Analyze 12.0 (Biomedical Imaging Resource Mayo Clinic, Rochester, MN).

### 2.5 Histologic examination

After the micro-CT imaging process, the tibias were decalcified for 48 ​h at room temperature. The decalcified samples were dehydrated in a graded ethanol series and embedded in paraffin. 5 ​μm thick sections were prepared and stained using hematoxylin and eosin, Masson Trichrome, and Saf-O, and counterstained with Fast Green. The sections were examined and photographed using Olympus Cooperation with CS-ST-V1 (cellSens Standard 1.11) software. A quantitative evaluation using histomorphometry was performed for the hole defect model. Histomorphometry measurements were quantified using the ImageJ software (National Institutes of Health, USA) and performed by a blinded investigator (S. L.). To evaluate newly formed bone, the sagittal-sectional area of the new bone, residual graft material, and connective tissue was measured [10,32]. The sample size was based on the recommendations of the IACUC and there was a minimum quantity of 3 rats per group. A Kruskal–Wallis test with post-hoc test (Dunn's multiple comparison) was conducted using Prism9 (GraphPad Software, San Diego, CA, USA) and the significance was set as *p* ​< ​0.05.

## 3 Results

### 3.1 Radiologic evaluation of critical-sized defect of the tibia

**Figure 5:**
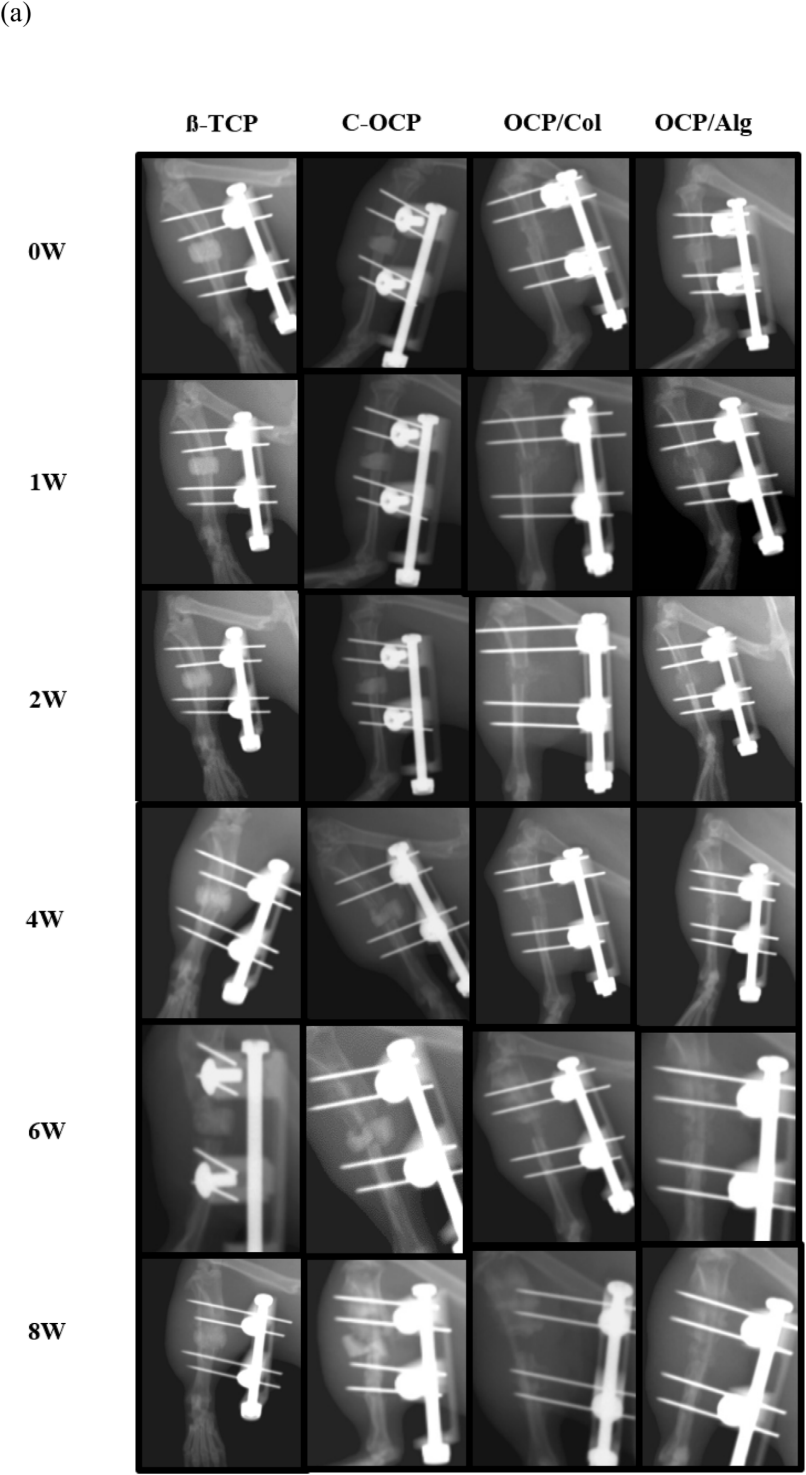

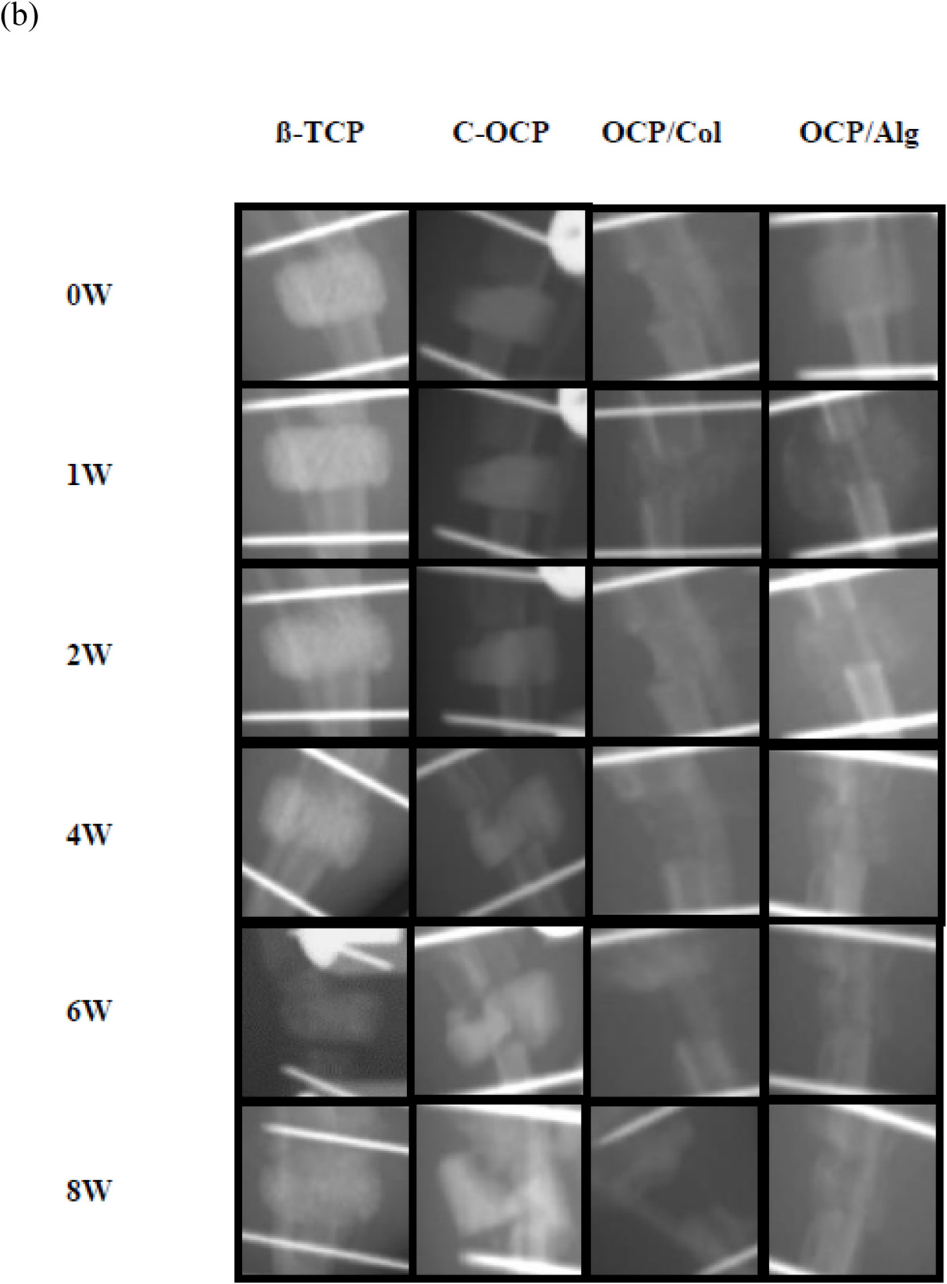

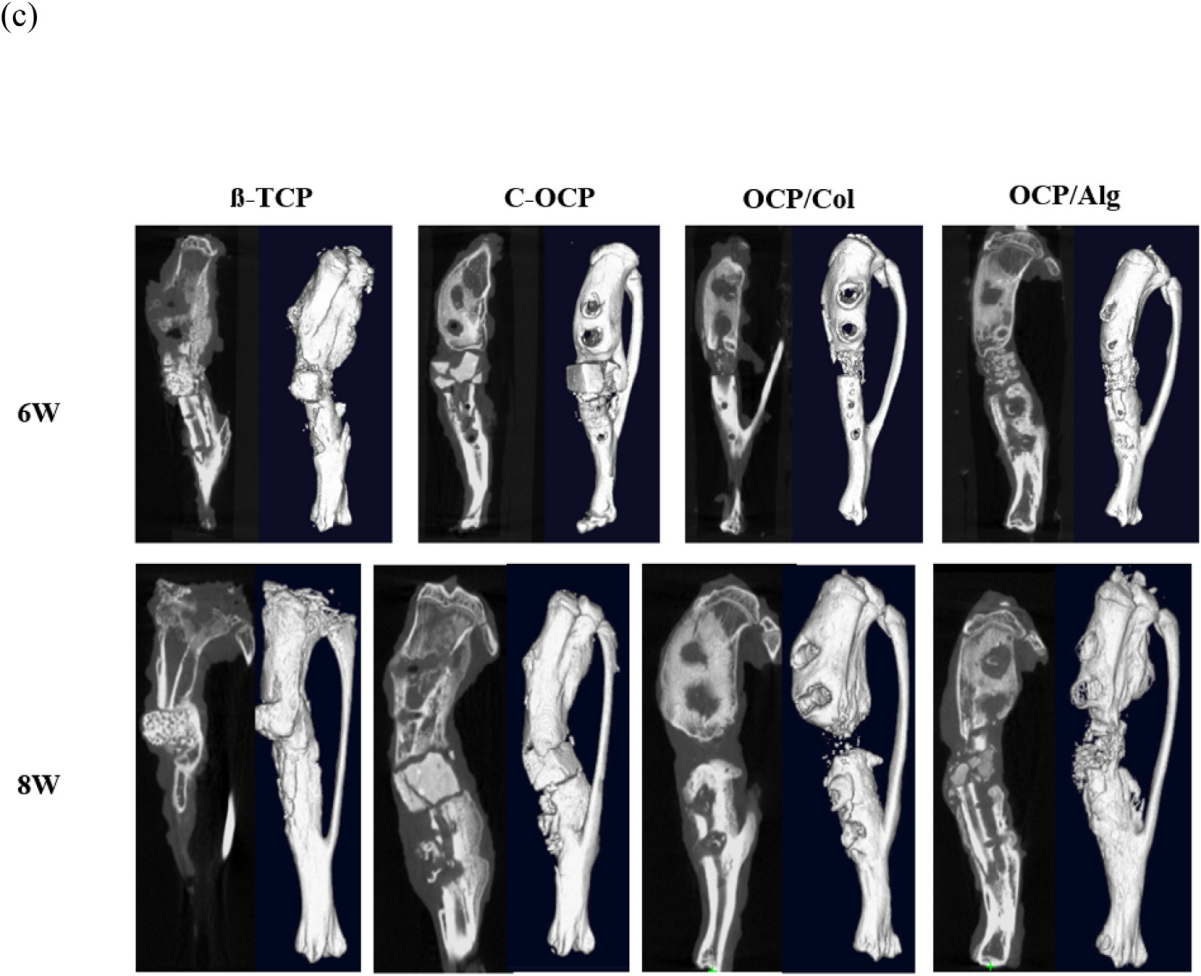
(a) Sequential radiographic sagittal view. (b) Magnified view (c) Micro-CT images of rat tibia at 6 and 8 weeks after implantation of β-TCP, C-OCP, OCP/Col and OCP/Alg.

The dual-energy X-ray absorptiometry (DEXA) scout view was serially evaluated from postoperative day (POD) 0 to the 8-week follow-up (Fig. 5(a)). In the immediate postoperative images, the scaffolds were observed to be stably fixed between the bone ends in each group. There were still definite specimen materials in the 8-week scan in the TCP and C-OCP groups. In the OCP/Col group, the POD 0 to 4-week scans demonstrated lesser radiopacity of the scaffold than the other three groups. At the 8-week scan, the external fixator appeared unstable, and there was minimal bridging bone formation. In the OCP/Alg group, the scaffold was clearly radiopaque in the POD 0 image and became less radiopaque up to the 4-week scan period. At the POD 6- and 8-week scans, the radiopacity was preserved and bridging bone formation was observed (Fig. 5(b)). Sample sagittal and 3-D micro-computed tomography (micro-CT) images of each group at the 6- and 8-week time points are shown in Fig. 5(c). The TCP group demonstrated bone ingrowth of the scaffold and bone formation along the posterior cortex. The C-OCP group did not demonstrate bony ingrowth into the scaffold and there was little radiographic evidence of degradation of the C-OCP scaffold. Some of the C-OCP scaffolds were dislodged from their original insertion sites, whereas others were fractured. In the OCP/Col group, spotted densities were observed in the POD 6- and 8-week images; however, it was unclear whether these densities were residual specimens or induced bone. Most of the tibias in the OCP/Col group demonstrated a decrease in the gap due to instability. In the OCP/Alg group, bony union was observed in the 6-week sagittal and 3-D images. In the β-TCP group, there was a bony bridge at the posterior cortex of the two bony ends from POD 6 weeks. The β-TCP material was still observed from the initial morphology until POD 8 weeks. In the C-OCP group, the C-OCP was maintained and was broken at POD 6 weeks and there was no bony union. In the OCP/Col group, the graft material was almost completely dissolved, and there was no radiopacity at POD 8 weeks. The critical gap decreased due to instability, and there was no bone bridge between the gaps. In the OCP/Alg group, the graft material was maintained but relatively dissolved compared to the β-TCP or other OCP groups. A bone bridge was observed from POD 6 weeks.

### 3.2 Histologic evaluation of critical-sized defect of the tibia

**Figure 6:**
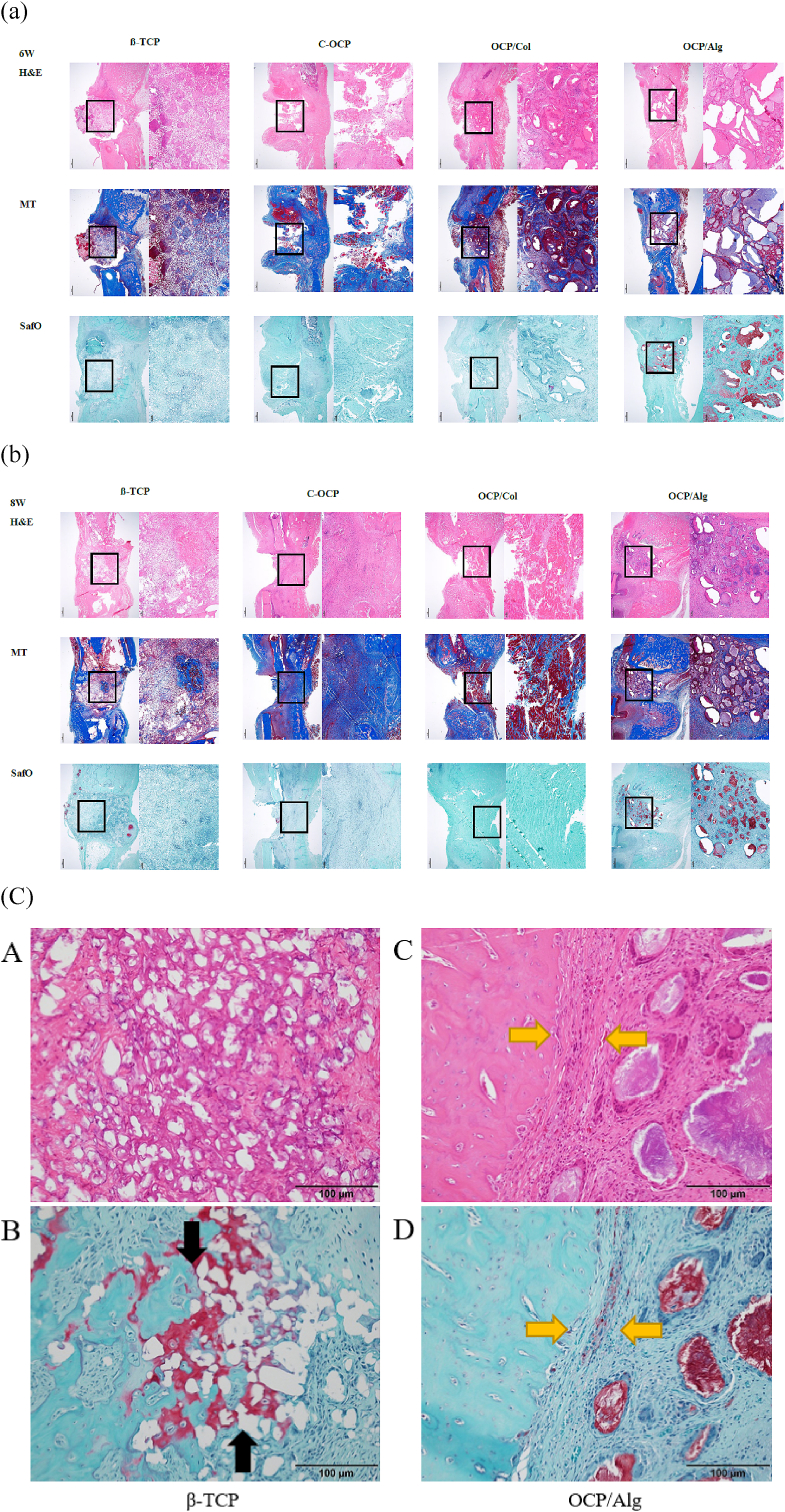
(a) Histological overview at postoperative 6 weeks. .Scale bars in the panels represent 1 ​mm. Left image, 12.5x right image, 40x (b) Histological overview at postoperative 8 weeks. Scale bars in the panels represent 1 ​mm. Left image, 12.5x; right image, 40x (c) Different appearance in new bone formation patterns between β-TCP and OCP/Alg materials from a histological point of view. Calcification in the β-TCP group occurred at the directly contacted area between graft particles (A). Also, bony ingrowth was observed in the region adjacent resected surface of tibia (B, black arrow). However, since the amount of bony ingrowth was small, most of the critical-sized defect was filled with retained grafted β-TCP and surrounding calcification. In contrast, no direct bony ingrowth was observed in OCP-based graft materials, but osteogenesis induced from resected surface of tibia was more active. Moreover, the defect gap was more reduced by new bone formation from the resected bone. Note the newly formed bone and grafted materials were separated by fibrous tissue (C and D, yellow arrow) (Original magnification: x200, and scale bar size: 100 ​μm).

In the β-TCP group, the grafted materials remained almost the same as when it was implanted, and a foreign body reaction was observed around the small graft material particles. New bone formation was observed from the cut surface of the tibia, and bony ingrowth from tibia into the graft materials was observed in the contacted area with the graft materials. Additionally, at POD 8 weeks, calcification surrounding grafted particles was observed although it did not show the mature shape of bone, and the foreign body reaction was alleviated. On the other hand, in the C-OCP group, a relatively large amount of graft materials was absorbed even at the POD 6 weeks, and it was replaced by fibrosis. In the group of OCP/Col group, it was observed that a large amount of grafted materials remained at both POD 6 weeks (Fig. 6(a)) and POD 8 weeks (Fig. 6(b)), and no bony ingrowth into the grafted materials was observed. However, at the resected surface of tibia, new bone was actively formed and the gap of the defect area was partially filled. In the OCP/Alg group, a relatively small amount of graft materials remained until the POD 8 weeks. As with other OCP-based materials, bony ingrowth into the graft materials was not observed; however, active new bone formation was observed at the resected surface adjacent to the graft material, filling the defect gap. In summary, in the β-TCP group, calcification around the graft particles and some bony ingrowth were observed while maintaining the transplanted shape. On the other hand, in the OCP-based group, the graft materials were partially absorbed and bone formation at the adjacent resected bone surface was observed. As a result, no bony ingrowth into the graft material was observed; however, the gap of defect was further reduced than β-TCP (Fig. 6(c)).

### 3.3 Radiologic evaluation of the hole defect of the tibia

**Figure 7:**
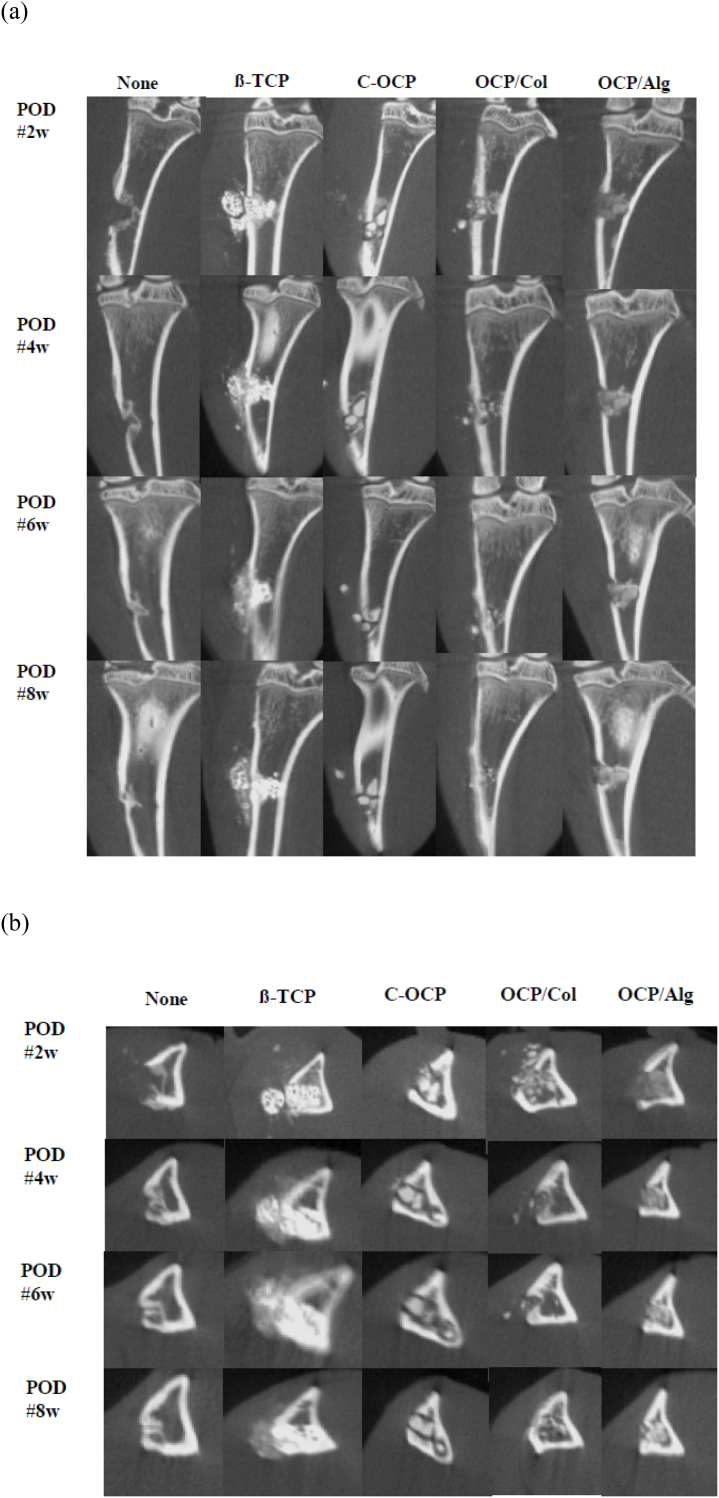
Serial micro-CT images of rat tibia. (a) Sagittal view (b) Axial view.

We serially evaluated micro-CT scans every other week, and the axial and sagittal cuts are shown in Fig. 7. In the negative control group (None), no materials were found in the hole to compare spontaneous bony union during the follow-up. At POD 6 weeks, there was spontaneous union; therefore, we further evaluated radiology between the groups to compare the bony union status of OCP composites. In the β-TCP group, bony union was observed during follow-up; however, most of the graft material that was inserted remained during follow-up. In the C-OCP group, the inserted OCP material was not biodegraded and was maintained in the hole compared with the negative control group (None) at POD 8 weeks. The 3-mm hole was almost united at POD 8 weeks without a bony substitute; however, graft materials in the C-OCP group consistently blocked bony ingrowth. In the OCP/Col group, the hole was almost united at POD 4 and completely united at POD 6. In the OCP/Alg group, there were residual OCP materials at POD 6 and 8 weeks; however, the radiopacity was similar to that of the adjacent bone, and bony union was observed to be different from that in the OCP group. Unlike the β-TCP group, the graft materials of OCP/Alg were observed to be biodegraded and were substituted with new bone formation during the follow-up.

### 3.4 Histologic evaluation of hole defects of the tibia and histomorphometric measurements

**Figure 8:**
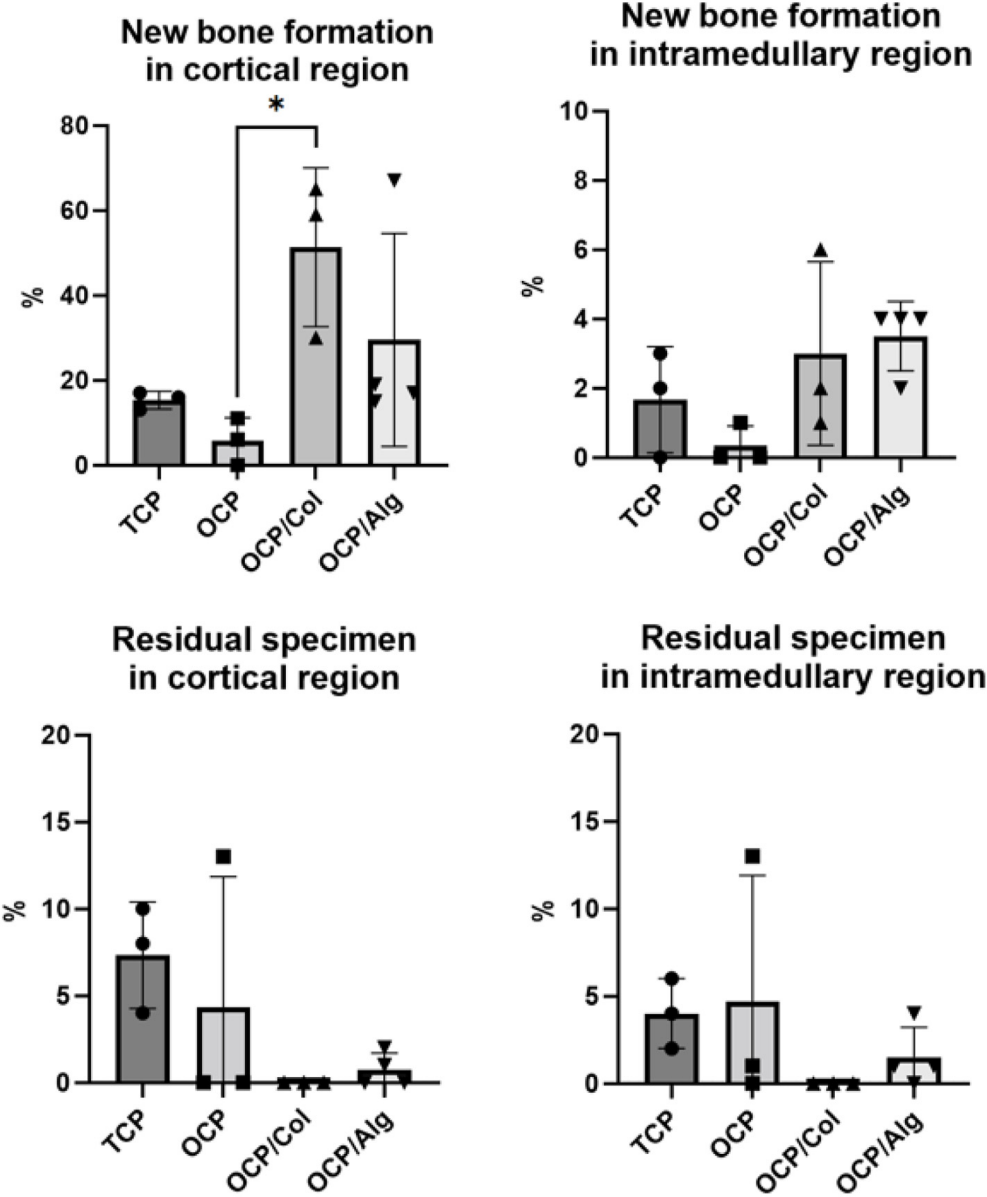
Histomorphometric analysis of newly formed bone area of total, cortical and intramedullary bone treated with β-TCP, C-OCP, OCP/Col and OCP/Alg in hole-defect model.

In the control group where the hole defect was observed to heal naturally, discontinuity of the cortical bone was observed at POD 4 weeks; however, cortical bone union was observed at POD 8 weeks. However, the thickness of the newly formed cortical bone was thin and the formation of cancellous bone was a small amount in the intramedullary region. In the case of the β-TCP group, calcification surrounding the graft materials was observed in the form of woven bone at POD 4 weeks, and the newly formed bony trabeculae became thicker at POD 8 weeks, having a more mature form of lamellar bone. However, it was observed that the tiny particles of graft materials escaped into the soft tissue outside the hole defect and induced foreign body reaction. On the other hand, in the OCP-based graft material, the bone formation in the adjacent area, not directly connected with grafted materials, was mainly observed rather than the bony ingrowth into the graft materials. Compared with the β-TCP group, the newly formed bone adjacent to graft material were cortical and thick trabecular bone, even at POD 4 weeks, and the cortical bone union was formed in defect site at POD 8 weeks. Only in the OCP/Alg group, direct calcification and osteogenesis surrounding the graft material particles were observed. As per the results of the critical defect experiment, it is presumed that OCP induces bone formation in the surrounding tissue, unlike β-TCP, in which calcification and osteogenesis occur directly surrounding the graft materials (Supplement Figure S2). New bone formation was statistically significant in the OCP/Col group compared to the β-TCP and OCP/Alg groups (∗*p* ​< ​0.05). OCP/Alg showed similar bone formation acceleration and better resorption of graft than β-TCP; however, the difference was not statistically significant (Fig. 8).

## 4 Discussion

As a bone substitute, calcium phosphate plays an important role in osteoconduction [33]. In contrast to β-TCP or calcium sulfate, previous studies have focused on OCP owing to its osteoinduction properties with relatively fast biodegradation [34,35]. A proper composite of materials or sintering process with OCP has not been demonstrated in preclinical studies with critical-sized defects in long bones. This study aimed to evaluate a newly manufactured OCP composite for use as a graft material in orthopedic surgeries. Among the various OCP composite materials in our study, β-TCP and OCP/Alg graft material showed effective bony ingrowth, and OCP/Alg showed better biodegradability than β-TCP at POD 8 weeks in both critical-defect and hole defect conditions. OCP/Col showed fast union in the hole-defect rat tibia model; however, early resorption was observed in the critical-sized defect tibia model. Because of this property, OCP/Col was not appropriate in our segmental defect model despite fast bone ingrowth. In contrast, the OCP-only composite material showed no biodegradability until POD 8 weeks in either the critical-sized or hole-defect tibia model, which is not acceptable as this will hinder bony ingrowth.

OCP is regarded as an in vivo precursor of HA with similar crystal structures. Previous studies have reported that OCP promotes osteoblast differentiation in vitro [13]. Studies have shown that the use of OCP as a bone substitute material is of interest from the point of view of osteogenesis in intramembranous ossification [36,37]; however, its clinical utility is still unclear and there is a lack of evidence compared to β-TCP or calcium sulfate, which are widely used in orthopedics. Regarding the phase composition, OCP has a two-layered structure of hydrate and apatite layers, and the apatite layers are involved in ion–exchange reactions at the phase boundary between the material and given environment [38]. Moreover, OCP has been proposed as a precursor of biological apatite in bones and OCP was reabsorbed by osteoclasts in addition to dissolution by pH in the body [36]. Recently, in vivo and clinical studies using OCP have shown that bone regeneration is superior to other predicate devices, such as synthetic and xenogeneic bones [39,40].

There are several aspects to consider for OCP composites to be commercially available graft materials. Unlike HA or β-TCP, the OCP phase cannot be maintained during sintering because of the large number of water molecules in the structure [41]. Therefore, the combination of OCP with other materials, such as polymers, is required to form implants. Second, OCP crystals should be dispersed within the matrix materials to initiate bone formation. Finally, the material properties should be maintained during storage under drying conditions and after sterilization. Several matrix material candidates were considered, and collagen and alginate were selected for our study.

Recently, OCP/Col has been demonstrated to enhance bone generation in several bone defect models. OCP/Col has already been proven to be safe in a clinical setting [42]; therefore, our study included OCP/Col for testing in orthopedic clinical situations. In contrast, mechanical stress loaded onto the OCP/Col graft induces excessive osteoclastic resorption, resulting in the complete resorption of the materials without bone regeneration [41]. In our study, the OCP/Col graft accelerated bony union in the hole-defect model without mechanical stress; however, it was rapidly degraded and showed rapid resorption in a segmental critical-sized defect model with weight-bearing. Therefore, OCP/Col was considered as an improper candidate graft material in a critical-defect rat tibia model. There was no inflammatory response, sign of infection, or other complications as a graft material; therefore, further study with alleviation of mechanical stress using a stress-shielding support material should be considered for application in clinical orthopedics.

Alginate is recognized as one of the useful materials that exhibit biocompatible, biodegradable, and implantable properties in vivo and works as a scaffold for active substances [43]. Fuji et al. showed that OCP-precipitated alginate provides a better scaffold for osteoblasts to attach and proliferate in a three-dimensional environment and promote bone regeneration, indicating that OCP is a candidate material to modify the surface of non-cell-interactive polymeric scaffolds, such as alginate, into osteogenic conditions [44]. Endo et al. suggest that OCP/Alg microbeads could be used as a vehicle to activate osteoblasts and deliver them to sites where bone regeneration is needed [43]. In our study, the OCP/Alg composite accelerated bony union comparable to commercially available β-TCP. β-TCP was maintained for a relatively long time compared to the OCP/Alg; therefore, OCP/Alg could be considered in relatively rapid biodegradable conditions and β-TCP would be appropriate in relatively maintained and slow conditions. Only the OCP/Alg specimen showed osteoblast induction by the adjacent bony end, as in previous studies [37,43,44].

Our study had some limitations. Our follow-up period was relatively short to evaluate mechanical stability, which is clinically important in the evaluation of bony union. After the 8-week follow-up, the bony union was not completed; therefore, there was no difference between the groups. Due to limitation of maintain rat external fixator for long time after 8 weeks, authors did not follow up further and added histologic analysis to see not complete union but acceleration of bone formation. In addition, because of the high variability of the specimens, quantitative measurements of micro-CT in the critical defect model were not conducted. TRAP or immunochemical histology was not used to evaluate the osteoclast or osteoblast activity.

## 5 Conclusions

In orthopedic theaters, β-TCP is used as a granule, rod, or wedge block bone form, depending on the surgical indication. Maintaining the β-TCP graft material until the completion of new bone formation is necessary in surgery, such as high tibial osteotomy or excision of benign tumors as simple bone cysts. Compared to β-TCP, OCP/Alg showed better biodegradability and osteoinduction properties; however, OCP/Col showed fast degradation, which is inappropriate for the clinical situation of weight-bearing. Therefore, OCP/Alg could be an option, depending on the clinical situation of relatively fast resorption and bony ingrowth.

## Authorship

All persons who meet authorship criteria are listed as authors, and all authors certify that they have participated sufficiently in the work to take public responsibility for the content, including participation in the concept, design, analysis, writing, or revision of the manuscript. Each author certifies that this material or part thereof has not been published in another journal, that it is not currently submitted elsewhere, and that it will not be submitted elsewhere until a final decision regarding publication of the manuscript in Journal of Orthopaedic Translation has been made. Indicate the specific contributions made by each author (list the authors' initials followed by their surnames, e.g., Y.L. Cheung). The name of each author must appear at least once in each of the three categories below.

Conception and design of study: Y.H. Kwak, K. Yun, C. Jeong, J. Kim; acquisition of data: S. Lim, J.H. Ji, D. Kim, A.M. Aldosari; Formal analysis: H.S. Kim, D. Han, A. Huser, S.B. Lee. Drafting the manuscript: C. Jeong, J. Kim, revising the manuscript critically for important intellectual content: Y.H. Kwak. Approval of the version of the manuscript to be published Y.H. Kwak, K. Yun, C. Jeong, J. Kim, S. Lim, J.H. Ji, H.S. Kim, D. Han, A. Huser, A.M. Aldosari, Y.G. Gim, S. B. Lee, D. Kim.

## Funding

This research was supported by grants from the National Research Foundation of Korea (NRF-2020R1F1A1075564), which is funded by the Korean government.

## Ethical approval statement

This study was conducted according to the guidelines of Yonsei University and was approved by the Animal Research Committee of Yonsei University (approval number 2020-0109).

## Article Processing Charge

The corresponding author agrees to pay the Journal of Orthopaedic Translation Article Processing Charge upon acceptance of the work for publication in Journal of Orthopaedic Translation, unless prior arrangements have been made to waive the Article Processing Charge.

## Declaration of competing interest

K.Y. is research director and J.K. worked on the manufacturer of specimen at HudensBio Co., Ltd. and all the other authors declared no competing interests.

## Appendix ASupplementary data

Figure S1 Surgery for the rat tibia hole defect model. After a 1 cm incision in the medial side of proximal tibia, a 1.2 mm sized K-wire was used to drill a pilot hole to prevent fracture while drilling with the larger bit. A 3 mm sized drill was used to drill the hole defect under saline irrigation to prevent thermal damage. The synthetic scaffold was implanted, and the incision was closed. Bilateral tibia were used and all rats were permitted to move freely right after surgery. Figure S1

Figure. S2 Histologic overview of tibia hole defect model. (a) At postoperative 4 weeks. Scale bars in the panels represent 1 mm. Left image is 12.5x magnification, right image is 40x magnification. (b) At postoperative 8 weeks. Scale bars in the panels represent 1 mm. Left image is 12.5x magnification, right image is 40x magnification. (c) Similar patterns of new bone formation with those in critical-sized defect was observed in hole defect experiment. Osteogenesis in the β-TCP group occurred at directly contacted area between graft particles, but trabeculae size of newly formed bone was thinner and more immature than t OCP-based materials. Among the OCP-based materials, directly contacted new bone was formed only in OCP/Alg group. The new bone formation in the periphery area of graft materials was much more active in the OCP-based materials, and the newly formed bone showed a thicker trabecular and more mature appearance than the β-TCP group. Note the directly contacted bone formation in β-TCP and OCP/Alg group (yellow arrow) and separation between newly formed bone and grafted materials in C-OCP and OCP/Col group (blue arrow). Figure. S2
