# Supplementary figures and images for: Acceleration of bone formation by octacalcium phosphate composite in a rat tibia critical-sized defect
Source: J Orthop Translat. 2022 Oct 12;37:100–12. doi: 10.1016/j.jot.2022.09.007 (PMC9574596; doi:10.1016/j.jot.2022.09.007)

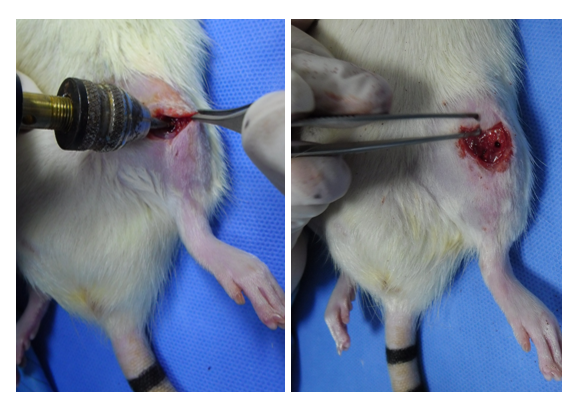
Supplement Figure S1

Supplement: Figure S1 — Surgery for the rat tibia hole defect model. After a 1 cm incision in the medial side of proximal tibia, a 1.2 mm sized K-wire was used to drill a pilot hole to prevent fracture while drilling with the larger bit. A 3 mm sized drill was used to drill the hole defect under saline irrigation to prevent thermal damage. The synthetic scaffold was implanted, and the incision was closed. Bilateral tibia were used and all rats were permitted to move freely right after surgery. [file mmc1.docx]

Supplement Figure S2

(a)


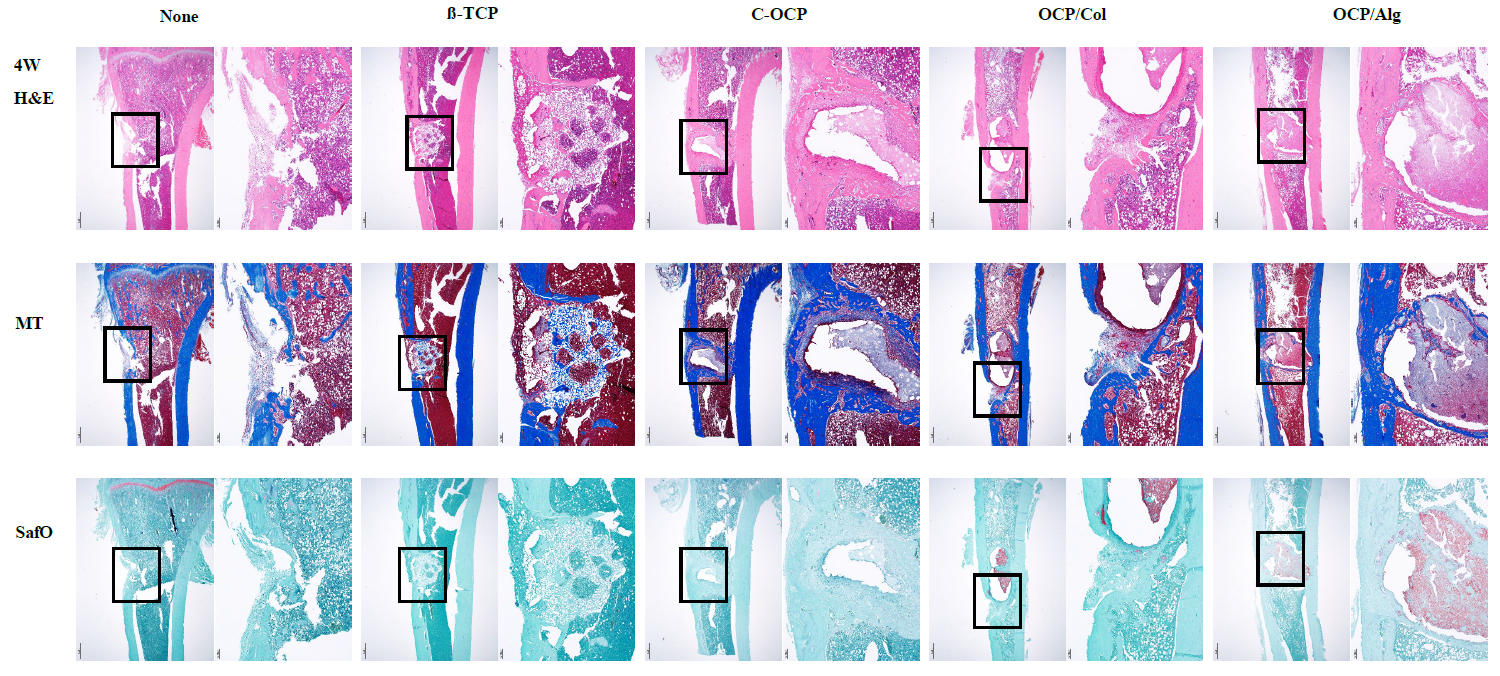


(b)


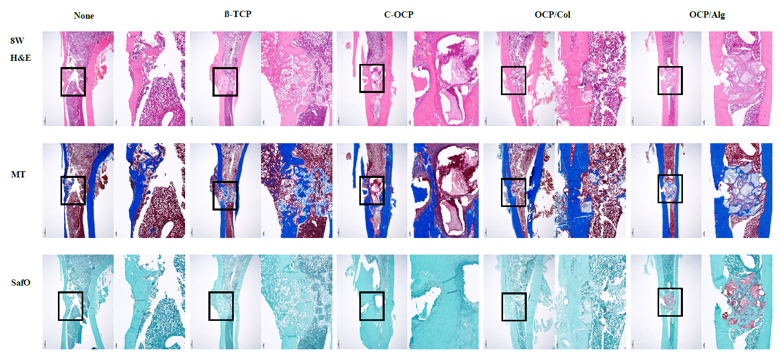


(c)


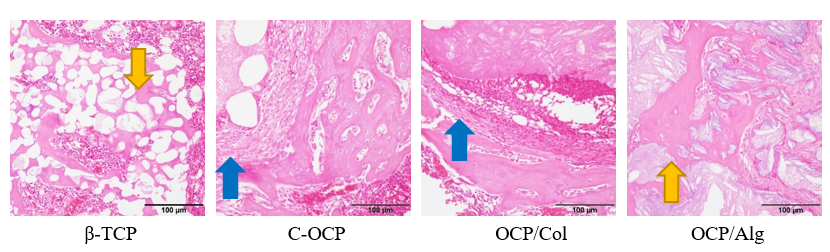

Supplement: Figure. S2 — Histologic overview of tibia hole defect model. (a) At postoperative 4 weeks. Scale bars in the panels represent 1 mm. Left image is 12.5x magnification, right image is 40x magnification. (b) At postoperative 8 weeks. Scale bars in the panels represent 1 mm. Left image is 12.5x magnification, right image is 40x magnification. (c) Similar patterns of new bone formation with those in critical-sized defect was observed in hole defect experiment. Osteogenesis in the β-TCP group occurred at directly contacted area between graft particles, but trabeculae size of newly formed bone was thinner and more immature than t OCP-based materials. Among the OCP-based materials, directly contacted new bone was formed only in OCP/Alg group. The new bone formation in the periphery area of graft materials was much more active in the OCP-based materials, and the newly formed bone showed a thicker trabecular and more mature appearance than the β-TCP group. Note the directly contacted bone formation in β-TCP and OCP/Alg group (yellow arrow) and separation between newly formed bone and grafted materials in C-OCP and OCP/Col group (blue arrow). [file mmc2.docx]
